# Research of Friction Stir Welding (FSW) and Electron Beam Welding (EBW) Process for 6082-T6 Aluminum Alloy

**DOI:** 10.3390/ma16144937

**Published:** 2023-07-11

**Authors:** Piotr Noga, Tomasz Skrzekut, Maciej Wędrychowicz, Marek St. Węglowski, Aleksandra Węglowska

**Affiliations:** 1 Faculty of Non-Ferrous Metals, AGH University of Science and Technology, A. Mickiewicza Av. 30, 30-059 Krakow, Poland; pionoga@agh.edu.pl (P.N.); skrzekut@agh.edu.pl (T.S.); 2 Faculty of Mechanical Engineering, Institute of Materials and Biomedical Engineering, University of Zielona Gora, Prof. Z. Szafrana Street 4, 65-516 Zielona Góra, Poland; 3 Łukasiewicz—Upper Silesian Institute of Technology, The Welding Centre, Bł. Czesława Str. 16–18, 44-100 Gliwice, Poland; marek.weglowski@git.lukasiewicz.gov.pl (M.S.W.); aleksandra.weglowska@git.lukasiewicz.gov.pl (A.W.)

**Keywords:** 6082-T6 aluminum alloy, FSW, EBW, microstructure, mechanical properties

## Abstract

The paper presents the results of the joining tests of the EN AW-6082 T6 alloy. The materials were joined using the EBW high-energy (electron beam welding) and friction stir welding (FSW) methods. In the case of FSW welding, the following parameters were used: the linear speed was 355 mm/min, and the rotational speed of the welding tool was 710. In the case of EBW welding, the following parameters were used: accelerating voltage U = 120 kV, beam intensity I = 18.7 mA, welding speed v = 1600 mm/min and, in the case of a smoothing weld, U = 80 kV, beam intensity I = 17 mA, and welding speed v = 700 mm/min. Comprehensive microstructural tests of all welded joints (MO, SEM and TEM) and mechanical property tests (tensile and hardness tests) were carried out. The topographies of the fractures after the tensile test were also examined. Based on the results, it was found that the strength properties of the EBW joint were reduced by 23% and the FSW joint by 38% compared to the base material. A decrease in elongation was also noted, with an FSW elongation of 7.2% and an elongation of 2.7% for EBW. In the case of the EBW joint, magnesium evaporation was found in the weld during welding, while in the FSW joint, the dissolution of the Mg2Si particles responsible for strengthening the material during heat treatment to the T6 state was observed.

## 1. Introduction

The production of semi-finished products or products from aluminum-based materials is most often carried out by plastic consolidation or casting. Aluminum alloys have unique properties such as a high tensile strength to density ratio, susceptibility to plastic consolidation, good corrosion resistance, etc. [1,2,3]. For this reason, they have found widespread use in such industries as the automotive industry (e.g., load-bearing components of trucks, car air-conditioning components), shipbuilding and the aviation industry [4]. Due to technological or economic limitations, various methods of joining aluminum alloys are also often used in manufacturing technologies.

Most of the problems associated with welding aluminum alloys result from its physical properties, such as high thermal conductivity, high chemical affinity for oxygen, a tendency to absorb hydrogen in the liquid state, high casting shrinkage and a linear expansion twice as high as in the case of steel [5]. These properties translate into, e.g., the formation of hard-to-melt oxides on the surface or the possibility of hot cracks [6,7]. Therefore, the main challenge faced by scientists is to determine the optimal connection process and the selection of parameters that will lead to a reliable connection. 

One of the most promising methods of joining aluminum alloys is the friction stir welding method. Friction stir welding (FSW) was patented in 1991 at the Welding Institute in Cambridge, UK [8]. This technology is considered one of the greatest achievements in the technology of welding materials in recent years and is still the subject of intensive research [9,10,11]. In the discussed method, the joining takes place below the melting point of the bonded materials, i.e., without the participation of the liquid phase. The amount of heat released in the FSW process is lower than traditional methods, which guarantees better properties of the joint. It is believed that the joint structure obtained is essentially devoid of defects often found in other methods [12]. The principle of the friction welding process with the mixing of the weld material consists of introducing a rotating tool with an appropriate shoulder and pin between the joined materials and moving it towards the contact line. The material flow around the pin is complex and depends in particular on the process parameters as well as the shape of the tool. The temperature difference across the cross-section of the joint, the plastic strain gradient and the strain rate cause different zones to form in the weld, which reflect the thermomechanical history of material flow [13]. Despite the local heterogeneity of the structure, fully recrystallized, equiaxed, fine grains are formed in the weld core as a result of intensive plastic deformation at elevated temperature. These small grains constitute a significant advantage of the FSW method over the other methods. Many years of experience and in-depth research into material flow in the FSW process have resulted in the significant development of tool designs. By using, e.g., spirals, grooves, grooves, protrusions or coaxial grooves on the surface, researchers improved the fit of the flange to the welded elements, reduced tool loads and increased the intensity of plastic deformation, especially at the joint surface. The importance of the flange participation in the welding process is also evidenced by the pressure force of the tool against the surface of the joined elements, which reaches values up to several kN [14,15,16]. In ref. [17], the authors determined the influence of shape on the microstructure and mechanical properties of joints welded with the FSW method from aluminum alloy of the 6082 grade. Three types of tools with different pin shapes and shoulder surfaces were used in the study. The process was carried out both unilaterally and bilaterally. It was shown that regardless of the tool used, high-quality welded joints were obtained without visible inconsistencies. The results obtained for the connection using a smooth shoulder without grooves with a flat flange are definitely lower. It was found that in double-sided welding, lower mechanical properties are obtained, which is related to the additional amount of thermal energy introduced into the material being welded in the second pass of the tool. It was found that in double-sided welding, lower mechanical properties are obtained, which is related to the additional amount of thermal energy introduced into the material being welded in the second pass of the tool. The change in mechanical properties was correlated with changes in the structure. In particular, a decrease in hardness in the nugget zone is associated with an intensive dynamic recrystallization. 

The second, constantly developing method of joining metals and their alloys is electron beam welding. Despite the fact that this technology has been known for over 60 years, in the last decade there has been a continuous increase in interest from both scientific units and industry. The electron beam welding (EBW) process consists of using the kinetic energy of electrons moving in a vacuum at a high speed (up to 200 km/s) [18,19,20,21]. The beam produced and formed in the electron gun goes to the area of the working chamber, where, falling on the contact areas of the joined objects, it melts it with heat obtained by bombarding it in a vacuum with a concentrated electron beam of high energy up to 10^12^ W/mm^2^. By adjusting both the electron beam and the operating mode of the electron gun (oscillating or stationary beam), we can regulate the amount of heat introduced to the connected elements in a wide range [22,23,24].

This article focuses on determining the quality of joints made of 6082 aluminum alloy, heat treated to the T651 condition. When selecting the alloy, its susceptibility to plastic processing was taken into account, and thus its common uses in industries such as the automotive (e.g., load-bearing elements of trucks) or shipbuilding industries was taken into account. At the same time, the limited weldability of the 6xxx series alloys, resulting from their tendency to hot cracking, and low fatigue strength force the search for new, more effective methods of joining them. In the global literature, one can find a large number of references to the subject of welding aluminum alloys of the 6xxx FSW and LBW series, often in comparison with traditional welding methods, such as MIG and TIG [25,26,27,28,29]. These methods are also applicable to steel [30]. However, there is no comparison of the properties of sheet joints made of this alloy in relation to modern, high-energy joining methods such as EBW. The purpose of this article is to analyze the FSW and EBW joints. The choice of these methods was dictated mainly by the discrepancies between the obtained welds in terms of shape, structure and properties. These differences are a direct result of the amount and concentration of energy supplied in a particular method. An analysis of mechanical properties was carried out using tensile and hardness tests. Microstructural analysis was performed using optical, scanning and transmission electron microscopy.

## 2. Materials and Methods

The material used for the tests was aluminum alloy 6082-T651 in the form of a sheet with dimensions of 6.35 mm × 1000 mm × 2000 mm. Solution heat treatment was carried out at a temperature of 540 °C, artificial aging at a temperature of 170 °C for 8 h and annealing by a controlled value (permanent deformation of 0.5% to 3%). The chemical composition of the 6082 alloy is shown in Table 1, and the mechanical properties in Table 2. Test joints were made on sheet sections of 150 × 300 mm. The welding process was performed along the edge parallel to the sheet’s rolling direction. Welding processes were performed at Łukasiewicz Research Network—Institute of Welding in Gliwice (currently Łukasiewicz Research Network—Upper Silesian Institute of Technology, the Welding Centre).

The friction welding process was carried out using the Triflute type tool. The welding linear speed was 355 mm/min, the rotational speed of the welding tool was 710 rpm, while the direction of rotation of the tool was clockwise. Electron beam welding was performed on a welding machine that was manufactured by Cambridge Vacuum Engineering. The following parameters were used: accelerating voltage U = 120 kV, beam intensity I = 18.7 mA, and welding speed v = 1600 mm/min. In addition, the smoothing joint was obtained with the following parameters: accelerating voltage U = 80 kV, beam intensity I = 17 mA, and welding speed v = 700 mm/min.

The tensile and bending tests of the obtained joints were carried out on the ZWICK ROEL Z050 testing machine. The width, thickness and length of the measurement base for the tensile test samples were 25 mm × 6.35 mm × 60 mm. The dimensions of the samples for testing the mechanical properties were selected on the basis of the PN-EN ISO 4136:2022-12 standard [32]. The Shimadzu HMV g hardness tester was used for hardness testing. Measurements were made with the indenter loaded with a force of 19.61 N (HV 2) for 10 s. The indentations were made in four measurement lines that passed through the native material, the sample weld and the heat-affected zone (HAZ) of each sample. Hardness measurements made in this way were used to prepare 3D maps in the MATLAB program and hardness profiles in welded joints.

The studies of the microstructure of the joints in the light microscopy (MO) mode were carried out on the Olympus GX51 light microscope (Tokyo, Japan). Test specimens were excised and cold mounted in Struers EpoFix epoxy resin. The samples prepared in this way were ground on abrasive papers with a grain size of 240–2000. Diamond suspensions were used for polishing, with a DP-Suspension P of 9 μm, 3 μm, and 1 μm, and the specimens were made on a RotoPol-11 grinding and polishing machine with a RotoForce-1 Struers head. Finishing polishing was carried out using the OP-S suspension from Struers (Copenhagen, Denmark). The last stage in the case of observing the samples in polarized light was anodizing in a solution of 1.8 mL HBF4 and 100 mL H_2_O using a voltage of 25 V for 30 s. The observation of the microstructure of the joints in scanning electron microscopy (SEM) and transmission electron microscopy (TEM) modes was performed on the HITACHI SU-70 electron microscope (Tokyo, Japan) equipped with a Thermo Scientific EDS system (Waltham, MA, USA). Chemical composition is given in weight %. Thin films for TEM observation were prepared by mechanical thinning on abrasive papers and the electrolytic polishing of mechanically cut samples in the Struers A2 reagent.

## 3. Results and Discussion

### 3.1. Microstructure

**Figure 1:**
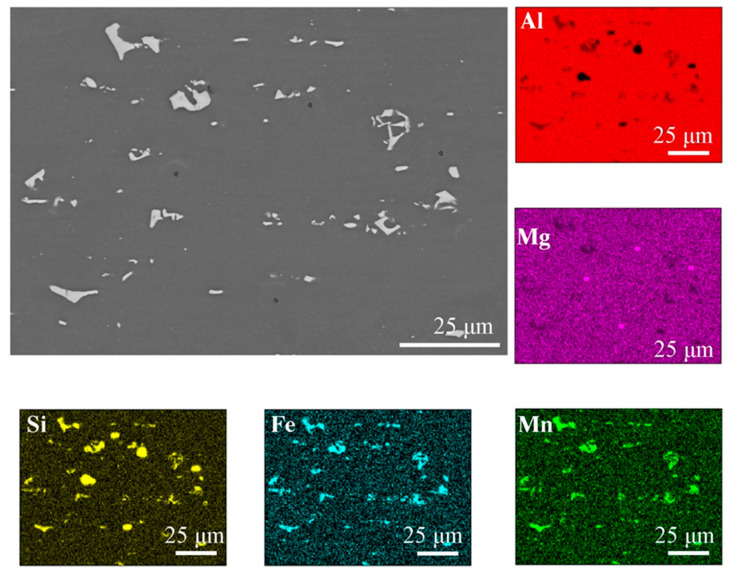
The microstructure of base material with the EDS chemical mapping.

Figure 1 shows the microstructure of the base material of the 6082 alloy (after solutioning and being artificially aged) in the direction transverse to the rolling direction. The average grain diameter, measured by the planimetric method, was 92 μm. Two types of phases can be distinguished: those containing magnesium and silicon (dark precipitates), and those containing aluminum, iron, manganese and silicon (light precipitates) shown in Figure 1. The element distribution map (Figure 1) confirmed the presence of the given chemical elements in the individual phases. Based on the chemical composition and literature analysis [33,34], it can be concluded that the bright areas are in the Al(FeMn)Si phase. Literature data indicate that in this type of alloy, depending on the chemical composition, there are phases with different stoichiometric composition (e.g., Al9Mn3Si, Al5FeSi, Al(FeMn)Si). These phases may also be characterized by a different morphology (they may have a columnar, polyhedral structure and may also be in the form of the so-called “Chinese script”). Dark precipitates rich in Mg and Si (Figure 1) are in the Mg_2_Si phase, the presence of which can also be confirmed in various scientific and research articles [35,36].

**Figure 2:**
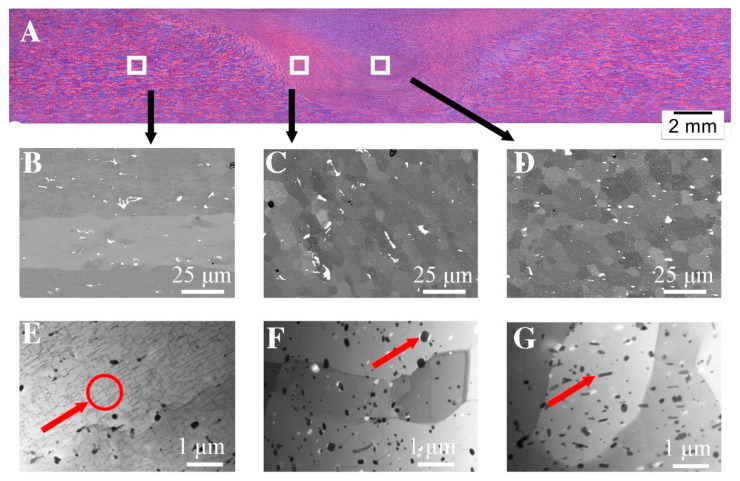
The microstructure of FSW joints: (**A**) light microscopy; (**B**) base material (SEM); (**C**) thermo-mechanically-affected zone (TMAZ), SEM; (**D**) nugget zone (NZ), SEM; (**E**) base material (TEM); (**F**) thermos-mechanically-affected zone (TMAZ), TEM; (**G**) nugget zone (NZ), TEM.

The microstructure of the FSW welded joint made of aluminum alloy grade 6082 (FSW 6082) visualized by light microscopy is shown in Figure 2A. Macroscopic examinations showed no inconsistencies in the obtained joint. In the lower part of the weld, where the mandrel had the greatest impact on the material, the nugget zone (NZ) can be distinguished, as well as the characteristic “onion rings” typical of many FSW joints of aluminum alloys [37]. In the analyzed joint, we can distinguish the advancing side and the retreating side. The advancing side shows a clear boundary that separates the thermoplastic deformation zone from the stir zone (SZ). This is due to the different relationship between the linear and rotational movement of the tool. On the retreating side, there is no clear boundary between the thermoplastic deformation zone and the stir zone. There is a gradual change in the microstructure from the centre of the weld to the thermo-plastic zone. More detailed differences in the microstructure in individual zones are shown in the tests carried out using a scanning electron microscope, shown in Figure 2B–D.

**Figure 3:**
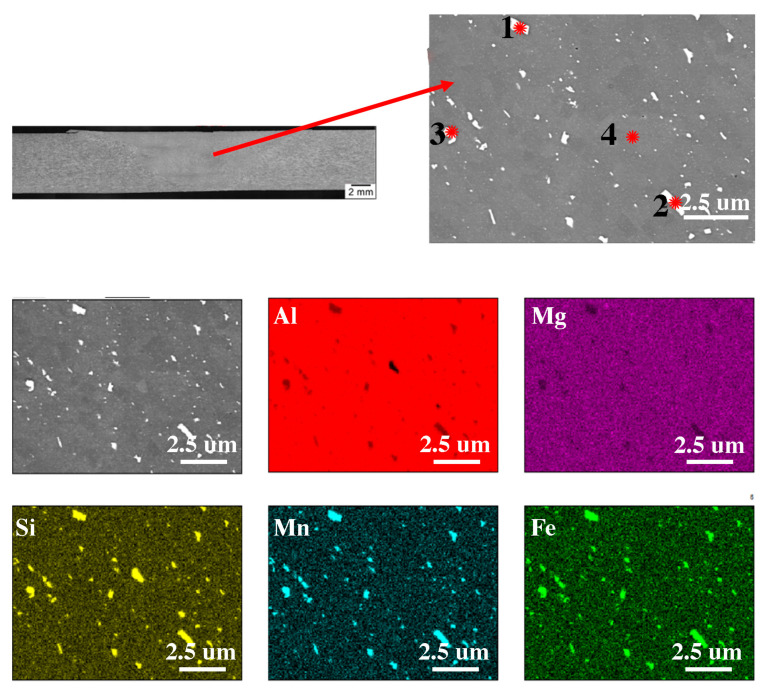
The microstructure in the cross-section of the FSW join with the EDS chemical mapping. EDS analysis results for grains marked 1, 2, 3, 4 are show in Table 3.

**Figure 4:**
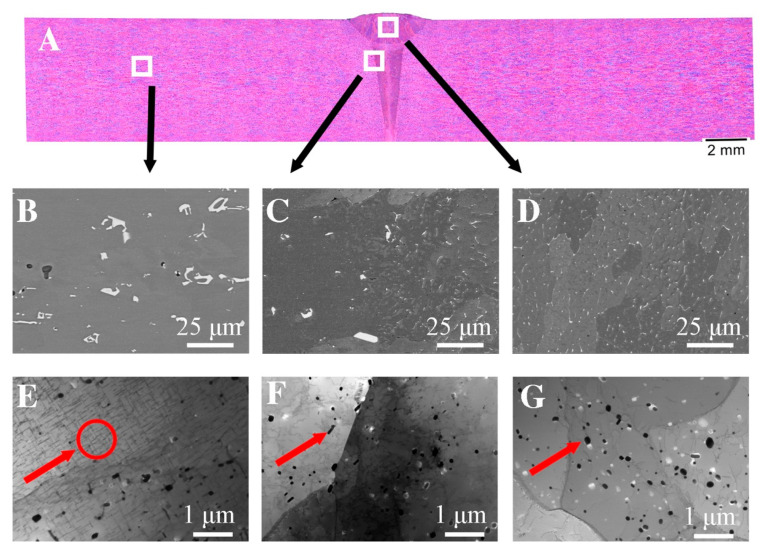
The microstructure of EBW joints: (**A**) light microscopy; (**B**) base material (SEM); (**C**) heat-affected zone (SEM); (**D**) weld zone (SEM); (**E**) base material (TEM); (**F**) heat-affected zone (TEM); (**G**) weld zone (TEM).

Figure 2B shows the microstructure of the base material, Figure 2C shows the thermo-mechanically affected zone (TMAZ) and Figure 2D shows the nugget zone (NZ). In terms of microstructure, the NZ shows the smallest diversity; it consists of recrystallized grains with an average diameter of 9 μm and particles of the Al(FeSi)Mn phases that were fragmented during the process, which is confirmed by the analysis of the chemical composition shown in Figure 3 and Table 3. Compared to the native material in the TMAZ zone, it can be observed that the grains on this side are elongated in the direction of material flow. In addition, as in the case of the nugget zone, spheroidization of the Al(FeSi)Mn precipitates can be observed. This leads to the minimization of the surface energy compared to the base material, which is confirmed by other scientific works [38]. This shape significantly reduces the stress concentration (accumulation) at the tops of the precipitates. In order to check the influence of deformation and thermal effects caused during the process, microstructural tests were performed using TEM electron microscopy Figure 2E and Figure 4F,G). In the base material (Figure 2E), we can observe lamellar precipitates of the Mg_2_Si phase from the {100} family, formed during precipitation strengthening, and dispersoids of the Al(FeSi)Mn phases. The microstructure of the TMAZ zone is shown in Figure 2F. The microstructure in this zone is affected by both the temperature gradient and plastic deformation from the FSW tool. Compared to the microstructures shown in Figure 2E, the occurrence of single dislocations and dislocation walls can be observed. The dislocation density is closely related to the deformation and recrystallization processes that occur during the process [39]. The authors [40] confirm that in the area of the weld nugget, the density of dislocations is the lowest in comparison with other areas of the weld. The presented results indicate the participation of healing processes in the recovery of this part of the weld. The TEM microstructure of the nugget zone (Figure 2G) reveals precipitations inside the grains. The grains contain small spherical precipitates and larger elongated ones.

The size of these precipitates is approximately 100 nm. Similar to the work in [17], the presence of reinforcing phases was not revealed in the nugget zone. The welding process was carried out at a temperature above the solvus line, which, combined with strong plastic deformation, caused the dissolution of the strengthening phases and the dynamic recrystallization process, which resulted in a fine-grained microstructure in the nugget zone. The aforementioned fragmentation of the Al(FeMn)Si intermetallic phases in the NZ and TMAZ regions may be evidenced by the increase in the volume fraction of fine dispersoids compared to the base material. An image of the microstructure of an electron beam-welded joint made of alloy grade 6082 (EBW 6082) made by light microscopy is shown in Figure 2A. Compared to the joint obtained by the FSW method, the EBW joint is characterized by a smaller weld area of 9.81 µm^2^ as well as a smaller average width of the heat affected zone—about 1 mm. Microstructural observations indicate the heterogeneity of the structure in individual areas of the weld caused by significant differences in the amount of heat input between different welds. In the smoothing joint, where the amount of linear energy was greater than in the area of the root of a weld, a dendritic structure with an average grain diameter of 151 μm is visible. A large temperature gradient caused the nucleation and growth of columnar grains. In the weld (Figure 3D), where the amount of heat supplied was much lower, equiaxed grains of the α solution with an average grain diameter of 42 μm with very fine precipitations of complex Al(FeSi)Mn phases were observed. The conducted SEM studies (Figure 4B–D), with the use of higher magnifications, indicate that these precipitates were significantly fragmented in relation to the base material. Due to the high cooling rate, the microstructure has changed both the morphology and dispersion of the Fe phases (white precipitates). The increase in the cooling rate slowed down the diffusion processes, which contributed to a significant reduction in the growth rate of phases rich in Fe, Si and Mn [41,42].

**Figure 5:**
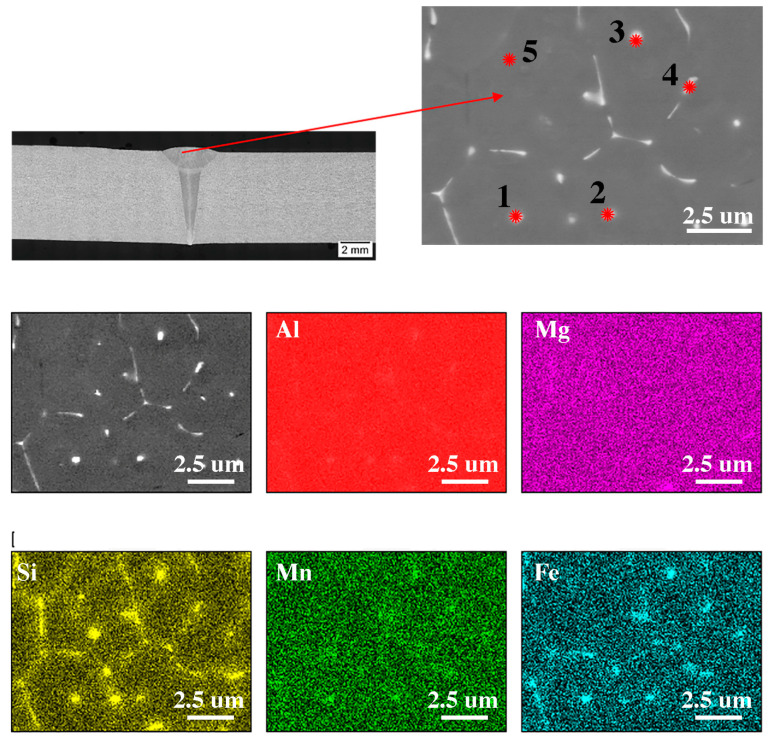
The microstructure in the cross-section of the EBW join with the EDS chemical mapping. EDS analysis results for grains marked 1, 2, 3, 4, 5 are show in Table 4.

The analysis of the chemical composition presented in Figure 5 and Table 4 confirm the presence of Al(FeSi)Mn phases located at the grain boundary and do not confirm the presence of magnesium-rich phases. In the native material (Figure 2E) the presence of plate-like precipitates of the Mg_2_Si phase and the dispersoid of the Al(FeSi)Mn phases was presented. In the area of the weld, there were no occurrences of Mg_2_Si lamellar precipitates; however, fine dispersoids of the Al(FeSi)Mn phases could be observed. Compared to the microstructure obtained in the FSW joint, a smaller amount of these particles can be seen, which are evenly distributed over the entire analyzed surface. In the case of the EBW joint, the average diameter of these particles was 110 nm, while in the FSW junction, it was 138 nm.

**Figure 6:**
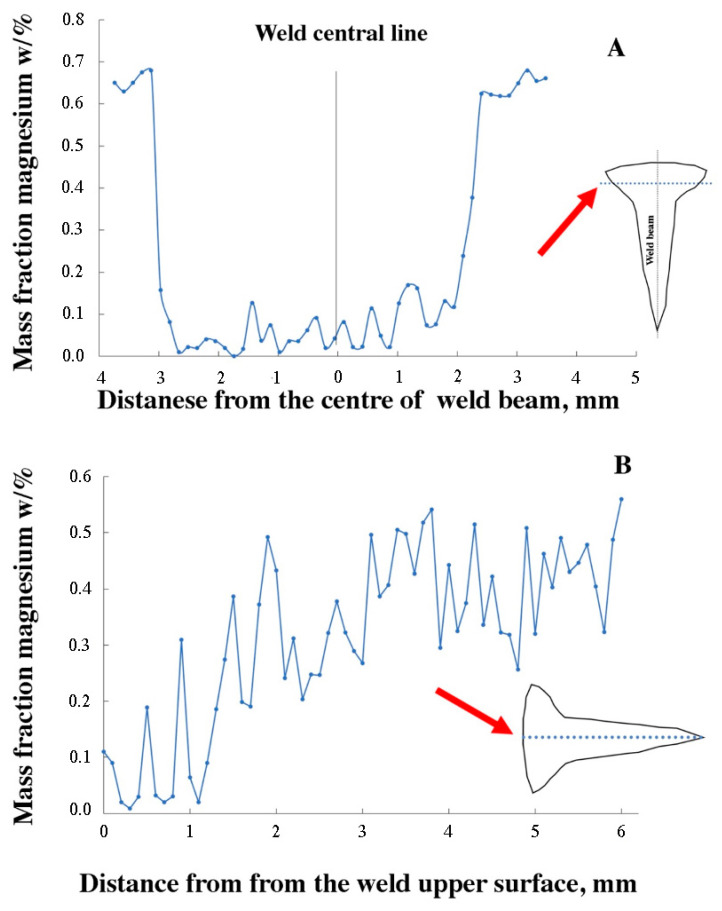
Mass fraction of magnesium (**A**) vertical to weld center, (**B**) along weld center line.

Due to the fact that phases rich in magnesium were not detected in the EDS analysis of the weld presented in Figure 5, Table 4, a linear analysis of the magnesium content in the weld was carried out in the cross-section (Figure 6A) and so was a longitudinal analysis (Figure 6B). The analysis showed a significant decrease in magnesium in the weld area compared to the solid material where the magnesium content was about 0.5% by mass. The lowest magnesium content was recorded in the upper part of the smoothing joint. The decrease in magnesium content is due to the low boiling point of magnesium. Magnesium sublimes from the weld in the form of fumes because the EBW welding temperature can reach about 3000–4000 K, which is well above the boiling point of magnesium of 1380 K [43,44,45]. The authors in [46] found that the vaporization of magnesium, apart from the low boiling point, is also caused by the thermal diffusivity and is closely related to the latent heat of vaporizing and melting of the materials.

### 3.2. Mechanical Properties

**Figure 7:**
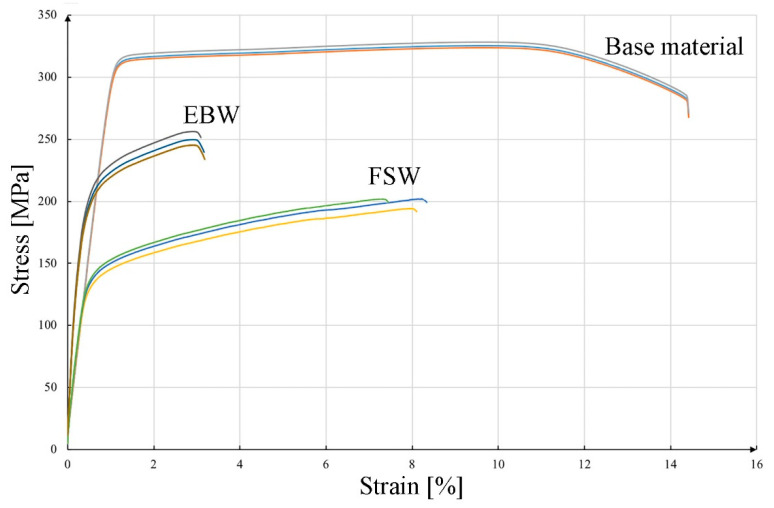
Stress–strain curves of aluminum: base material, FSW joint, EBW joint.

The results of mechanical property tests for the base material, FSW and EBW joints are shown in Figure 7 (curves: engineering stresses and engineering strains) and Table 4. The tensile strain rate and temperature when testing the samples of welded joints were selected on the basis of the PN-EN ISO 4136:2022-12 standard. The base material obtained the highest strength and plastic properties. The average tensile strength of the parent material was 325 MPa, while the yield strength and elongation were, respectively, 302 MPa and 13.1%. The achieved results meet the requirements of the standard [31] and are close to the values available in the literature for 6082 in the T6 state [47]. The tensile strength of the EBW joint was 250 MPa, while the yield strength and elongation were 210 MPa and 2.7%. The decrease in elongation compared to the base material and the FSW joint is most likely due to the welding conditions during the process. Rapid cooling resulted in the location of precipitates rich in Fe, Si, Mn on grain boundaries; additionally, these precipitates have morphologies with sharp edges, which may propagate cracks. A similar decrease in mechanical properties was noted by the authors in the case of welding the 6156 alloy in the T6 state [48]. The joint made using the FSW method had a tensile strength of 223 MPa, and the yield strength and total deformation were 150 MPa and 3.2%, respectively. Similar properties of tensile strength were obtained by Krasnowski [17], but in this case, the authors compared the mechanical properties and microstructure of FSW joints depending on the shape of mandrels used to join sheets made of alloy 6082. In the work of Svensson [49], the tensile strength of FSW joints was comparable for a 10 mm thick sheet. The obtained strength properties for both joints met the standard for the minimum strength of aluminum welded joints [50].

**Figure 8:**
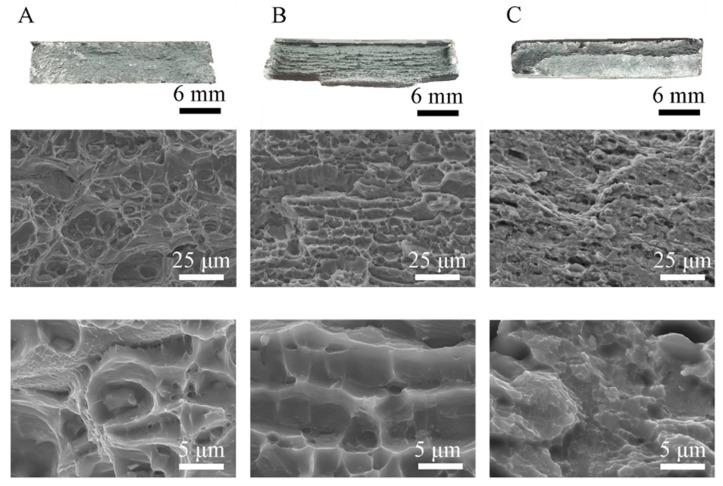
Fracture topography of 6082-T6: (**A**) base material, (**B**) FSW joint, (**C**) EBW joint.

Figure 8 shows fractures of the samples after the tensile test. Figure 8A shows the fractures of a sample of the base material. The observations indicate that the tested alloy shows a ductile nature of cracking, as evidenced by numerous micro holes on the fracture surface. The plastic nature of cracking is confirmed by the test results obtained (Figure 7, Table 5). A characteristic feature of the examined fracture are particles of the second phase, which are very often located at the bottom of the microwells. These particles, shown in Figure 1, appear as bright phases. The location of the particles in the microwells may indicate the propagation of the cracking process on these particles. The fracture of the sample after stretching from the FSW joint is shown in Figure 8B. The joint has broken in the heat-affected zone. The morphology of the fracture shows the parallel arrangement of the microwells in the direction of the stem mandrels in the case of the fracture of the sample from the native material. Also, in this case, numerous microwells with particles of the second phase are located at the bottom. The micro holes formed during the tensile test have a spherical shape in most cases and a similar size throughout the fracture. The particles of the second phase are much smaller than observed in the base material. Figure 8C shows the fracture obtained with the EBW method. The analysis showed no welding imperfections in the observed fracture, and the joint broke in the heat affected zone. This fracture (Figure 8C) is a typical example of a brittle fracture, as cleavable planes with sharp edges can be seen, which can be related to the results of plastic properties shown in Figure 7 and Table 5. The low plasticity in the EBW joint and the fracture of the brittle fracture type are caused by the unfavorable arrangement of the precipitates at the grain boundary. However, in the case of the fracture of the FSW joint and the base material, the fractures were plastic, which translated into a higher elongation obtained in the tensile test.

**Figure 9:**
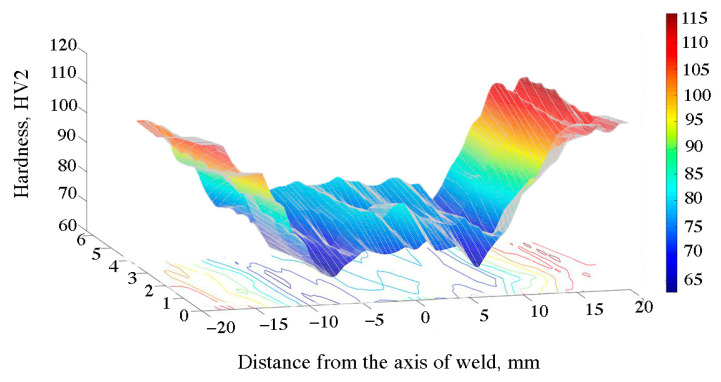
Hardness distribution map of the FSW joint.

**Figure 10:**
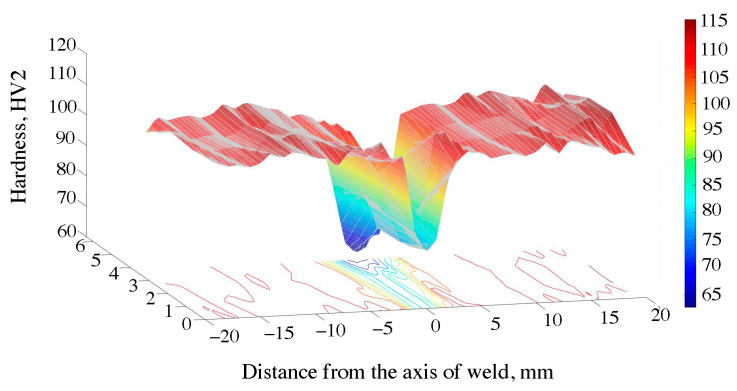
Hardness distribution map of the EBW joint.

The hardness distribution map for the FSW joint is shown in Figure 9 and in Table 6. The curve consists of the central area, parallel to the abscissa axis, which corresponds to the width of the weld. The hardness in this section amounted to approx. 78 HV and, compared to the base material, it decreased by 26%. The difference in the hardness between the joint area and the base material is caused by the action of deformation and high temperature during the process. As a result, the strengthening phases were dissolved and the microstructure was changed by dynamic recovery and recrystallization, as evidenced by the microstructure shown in Figure 4. The lowest hardness was recorded at a distance of approx. 10 mm from the weld axis, in the area of the heat affected zone. At this point, the hardness decreased by approx. 35 HV compared to the native material and 10 HV in the case of the mixing zone. A similar dependence on hardness changes in material 6082, subjected to the process of solutioning and aging, was noted by other authors [51,52,53]. The hardness distribution map for the EBW 6082 joint is shown in Figure 10 and in Table 6. In this case, the hardness profile for the EBW joint resembles the letter “V”. The center of the weld was characterized by the lowest hardness, the value of which was 72 HV. The decrease in hardness of 35% in this area was caused by the evaporation of magnesium, as shown in Figure 6A,B. The average hardness in the HAZ was approx. 90 HV. It can be seen that in the case of the EBW joint, the hardness in the HAZ was higher than in the case of the joint obtained by the FSW method. Differences in this zone are most likely due to differences in the amount of heat supplied during the joining process. The authors in papers [54,55] explain the decrease in hardness in the HAZ zone in FSW joints with the high temperature during the joint process and the large volume of plasticized material. The decrease in hardness in this zone is caused by the dissolution of the strengthening phases (formed as a result of supersaturation and aging to the T61 state).

The test results of various welding methods of aluminum alloy 6082 presented in the paper indicate a significant impact of the joining method on the properties of the joint. The main factor causing structural differences, and thus changes in the properties of joints, is the effectiveness of the impact of heat generated as a result of the energy supplied in the welding method used, causing structural changes. The FSW-welded joint studied in this paper is of great interest due to the possibility of joining metals and alloys that are difficult to weld using other methods [56,57]. The FSW process is environmentally friendly and the energy expenditure is comparable to conventional methods. This technology makes it possible to obtain good quality welded joints, which was confirmed by the results of the research carried out in this work. The EBW-welded samples, where the cooling rate was higher compared to the FSW method, are characterized by an elongation of 2.7%, and the fracture after the tensile test was characterized by a brittle fracture. Thermal stresses arose in the welded joint, which in the areas of tensile stresses led to the cracking of the joints, which was also not favored by the unfavorable arrangement of the precipitates at the grain boundary.

## 4. Conclusions

The applied joining technologies of the 6082 aluminum alloy allowed us to obtain homogeneous joints without inconsistencies and with comparable mechanical properties. The joint obtained by the EBW method obtained a tensile strength of 76% in relation to the starting material, while the FSW joint was 61%.

The low plasticity in the EBW joint and the fracture of the brittle fracture type are caused by the unfavorable arrangement of the precipitates at the grain boundary. However, in the case of the fracture of the FSW joint and the base material, the fractures were plastic, which translated into a higher elongation obtained in the tensile test.

The microstructural analysis of the FSW joint showed that structure-renewal processes dominated in the weld area. There is a change in the morphology and dissolution of the particles due to high temperature and the influence of the tool.

In the joint obtained by the EBW method, magnesium loss was observed in the weld, which contributed to a decrease in hardness in this area. The largest loss of magnesium took place in the upper part of the weld, where an additional smoothing joint was made.

## Figures and Tables

**Table 1:** Chemical composition of 6082 alloy.

Element	Al	Si	Fe	Cu	Mn	Mg	Cr	Zn	Ti	Other
Required by EN 1706 for base metal	97.13	1.0	0.3	0.05	0.67	0.6	0.06	0.07	0.07	0.05

**Table 2:** Mechanical properties of base metal 6082 [31].

Element	UTS, MPa	YS, MPa	Elongation, %	Hardness, HB	Density, g/cm^3^
Required by EN 1706 for base metal	≥310	≥260	≥10	≥91	2.7

**Table 3:** Analysis of the chemical composition of the FSW 6082 joint in the area of the weld nugget, % by weight.

Nugget Zone (NZ)	Mg	Al	Si	Mn	Fe
**1**	0.0	60.7	9.9	15.6	13.8
**2**	0.0	59.0	8.4	13.8	18.8
**3**	0.0	61.6	8.7	12.4	17.3
**4**	0.5	97.9	1.1	0.2	0.3

**Table 4:** Analysis of the chemical composition of the EBW 6082 joint in the area of the weld, % weight.

EBW 6082_Face of Weld	Mg	Al	Si	Mn	Fe
1	0.0	85.0	3.6	3.1	8.3
2	0.0	84.3	4.9	3.8	7.0
3	0.0	84.9	4.0	3.6	7.5
4	0.0	85.8	3.9	3.0	7.3
5	0.4	97.8	1.2	0.3	0.3

**Table 5:** Mechanical properties of materials.

	Base Material	St. Dev.	EBW	St. Dev.	FSW	St. Dev.
UTS, MPa	325.00	2.17	250.00	5.42	200.00	5.05
YS, MPa	302	2.12	210	3.70	140.30	3.10
Elongation, %	13.10	0.7	2.70	1.60	7.2	1.20

**Table 6:** Hardness results of the analyzed joints.

	EBW	St. Dev.	FSW	St. Dev.
Base material	110	5.03	110	5.03
Heat affected zone	90	4.20	70	4.10
Weld	72	3.60	78	2.20

## Data Availability

Not applicable.
